# The Impact of Type 2 Diabetes Mellitus on Non-Surgical Periodontal Treatment: A Non-Randomized Clinical Trial

**DOI:** 10.3390/jcm13195978

**Published:** 2024-10-08

**Authors:** Lícia Clara Garcia Belizário, Carlos Marcelo S. Figueredo, João Victor Soares Rodrigues, Thamiris Cirelli, Rafael Scaf de Molon, Valdir Gouveia Garcia, Letícia Helena Theodoro

**Affiliations:** 1 Department of Diagnosis and Surgery, School of Dentistry, São Paulo State University (UNESP), Araçatuba 16015-050, SP, Brazil; liciabelizario@gmail.com (L.C.G.B.); joao.vic.t@hotmail.com (J.V.S.R.); rafael.molon@unesp.br (R.S.d.M.); leticia.theodoro@unesp.br (L.H.T.); 2 School of Medicine and Dentistry, Griffith University, Brisbane, QLD 4101, Australia; 3 Department of Dental Medicine, Karolinska Institutet, 171 77 Solna, Sweden; 4 Center for Dental Assistance to Persons with Disabilities (CAOE), School of Dentistry, São Paulo State University (UNESP), Araçatuba 16018-805, SP, Brazil; 5 Department of Dentistry, University Center of Associated School—UNIFAE, São João da Boa Vista 13870-377, SP, Brazil; thamiriscirelli@gmail.com; 6 Latin American Institute of Dental Research and Education (ILAPEO), Curitiba 80710-150, PR, Brazil; vg.garcia@uol.com.br

**Keywords:** diabetes mellitus, periodontitis, periodontal disease, scaling and root planing, glycated hemoglobin

## Abstract

**Background/Objectives:** Periodontitis (P), a chronic inflammatory condition that affects the supportive tissues around the teeth, is three to four times more prevalent in individuals with diabetes mellitus (DM), with a direct correlation between its severity and the levels of glycosylated hemoglobin (HbA1c). This study aimed to evaluate the periodontal clinical parameters following non-surgical periodontal treatment (NSPT) in P patients with or without type 2 DM. **Methods**: Forty patients with P were divided into two groups: Group DM/P and Group P. All the patients were assessed at baseline and at 90 and 180 days after receiving NSPT. The parameters evaluated included the HbA1c level, plaque index (PI), probing pocket depth (PPD), clinical attachment level (CAL), and bleeding on probing (BoP). A statistical analysis was performed with a significance level set at α = 5%. **Results**: There were significant differences in the HbA1c levels between the DM/P and P groups at baseline, 90, and 180 days, as expected. Importantly, the HbA1c levels did not change after NSPT. Group P showed a significant reduction in both the PI and the BoP values at 90 and 180 days (*p* < 0.05). In contrast, Group DM/P demonstrated a significant increase in the percentage of sites with a PPD ≥ 5 mm at 180 days (*p* < 0.05). Additionally, Group P exhibited an increase in sites with a PPD ≤ 4 mm and a decrease in sites with a PPD ≥ 5 mm at both 90 and 180 days (*p* < 0.05). **Conclusions:** Our findings suggest that DM may compromise the effectiveness of NSPT, potentially hindering favorable outcomes during the follow-up period.

## 1. Introduction

Diabetes mellitus (DM), a chronic metabolic disease, is characterized by either a partial or total deficiency in insulin production (type 1 DM) or a periphery resistance to insulin’s action (type 2 DM). This condition disrupts glucose, protein, and lipid metabolism, leading to hyperglycemia and multiple systemic abnormalities [1], in which periodontitis being considered the sixth complication associated with DM. By 2045, it is estimated that nearly 693 million people will have DM, with approximately 90% of cases being type 2 DM [2,3].

DM can be classified into several types, including type 1, type 2, gestational, and other specific types [4]. DM1 results from the destruction of β-cells in the pancreatic islets of Langerhans, leading to complete insulin insufficiency, which may be associated with autoimmune processes or have an idiopathic origin. DM2, on the other hand, is a heterogeneous syndrome caused by defects in both insulin secretion and action, with its pathogenesis linked to genetic and environmental factors [5]. DM2 is the most common form and typically progresses from insulin resistance (the reduced sensitivity of target tissues to insulin) to insulin deficiency due to secondary β-cell failure in the pancreas [1]. The incidence and prevalence of type 2 DM have been rising around the world, making it one of the most widespread diseases globally [6]. The symptoms of type 2 DM include polyuria (excessive urine production), polydipsia (increased thirst), weight loss that is sometimes accompanied by polyphagia (excessive appetite), and blurred vision. Obesity is a significant risk factor for the disease [7]. The blood glucose levels in DM patients are represented by ≥126 mg/dL of plasma glucose, while patients with values below 99 mg/dL are considered to be normoglycemic patients. Patients with values between 100 and 125 mg/dL are considered to be pre-diabetic patients [1].

Since 2009, glycosylated hemoglobin (HbA1c) has been used as a diagnostic marker for DM. HbA1c represents a fraction of the hemoglobin formed in the presence of hyperglycemia; thus, higher blood glucose levels result in a higher proportion of HbA1c [8]. The HbA1c test is advantageous because it provides an estimate of average blood glucose levels over the previous 60 to 90 days, unlike fasting blood glucose or glucose tolerance tests, which measure glucose at specific points in time. Monitoring HbA1c is essential for evaluating glycemic control and reducing the incidence of complications [9].

Periodontitis, a multifactorial chronic inflammatory condition associated with a dysbiotic bacterial plaque biofilm, is characterized by the progressive destruction of the tissues that support the teeth, including periodontal ligaments, cement, and alveolar bone [10,11]. According to the recent 2018 periodontitis classification, the periodontitis definition should include the loss of periodontal tissue support due to inflammation, i.e., the loss of interproximal clinical attachment level of ≥2 mm or ≥3 mm at two non-adjacent teeth [10]. Once periodontitis has been diagnosed, clinicians should identify the disease’s severity and its progression rate based on a framework of multidimensional periodontitis staging and grading [10]. The stages of periodontitis are mainly based on the amount of clinical attachment loss, varying from 1−2 mm (stage I), 3–4 mm (stage 2), and >5 mm (stage III and IV). The disease develops in response to bacterial presence and the associated toxins, triggering an immuno-inflammatory response [12,13]. This process involves the release of pro-inflammatory cytokines, which influence immune cell activity, differentiation, proliferation, and survival and also regulate the production and activity of other cytokines, either amplifying (pro-inflammatory) or attenuating (anti-inflammatory) the inflammatory response. Of importance, periodontitis has been linked to the aggravation of other non-communicable chronic conditions, such as cardiovascular disease [14,15], rheumatoid arthritis [16], DM [17,18], and nonalcoholic fatty liver disease [19].

The intensity of this response depends on both the pathogenicity of the microorganisms present and the host’s susceptibility, which can lead to the destruction of periodontal tissues [20]. The bidirectional relationship between diabetes and periodontitis has been extensively studied [21,22,23]. DM is a major risk factor for periodontitis, with studies indicating that the risk of developing periodontitis is approximately three to four times higher in patients with poor glycemic control compared to normal glycemic patients [24,25,26]. Both conditions are associated with changes in the immuno-inflammatory system, which are characterized by increased levels of pro-inflammatory cytokines, such as interleukin (IL)-1, IL-6, and tumor necrosis factor-α (TNF-α) [27]. These features are enhanced in type 2 DM and periodontitis, triggering a cascade of events that result in the greater destruction of periodontal tissues [27]. Patients with HbA1c values greater than or equal to 7% tend to present a greater development and progression of periodontitis [28]. Moreover, there is a direct relationship between the level of glycemic control and the severity of periodontitis [29]. Research suggests that DM reduces the collagen levels in periodontal tissues, hinders collagen synthesis, and increases the degradation of the connective tissue [30]. Additionally, in individuals with DM, proteins undergo glycation, leading to the formation of advanced glycation end products (AGEs). These AGEs have various cellular interactions, including altering macrophage function and stimulating the release of inflammatory mediators, such as growth factors and cytokines, which prolong the inflammatory response [31].

Non-surgical periodontal treatment (NSPT) interventions typically include oral hygiene instruction (OHI) and subgingival instrumentation (SI), sometimes combined with supportive therapies [32], such as chemical methods for plaque control. The “gold standard” for maintaining periodontal health is the mechanical removal of dental biofilm through conventional debridement, which is an effective approach to treating periodontitis [32]. SI is considered a fundamental and conventional therapy in periodontal treatment [33]. This approach reduces pathogenic microorganisms, decreases the probing pocket depth (PPD), reduces the bleeding on probing (BoP), and promotes clinical attachment level (CAL) gain [33]. SI effectively controls the inflammatory process and reduces the PPD in patients with periodontitis [32]. However, the method used in SI is not the most critical determinant of periodontal treatment success [34]. The success of NSPT depends on a combination of detailed root debridement, adequate periodontal maintenance therapy, and patient compliance [33].

In patients with both periodontitis and DM, SI has been found to influence the levels of AGEs, which play a key role in the pathogenesis of both conditions. SI has been shown to reduce the levels of AGEs in the periodontal tissues [9]. AGEs accumulate more rapidly in individuals with diabetes due to prolonged hyperglycemia [1]. They contribute to tissue damage by promoting oxidative stress and inflammation. By reducing periodontal inflammation through SI, the production of AGEs can be diminished, improving tissue health [9]. SI, by reducing periodontal inflammation, can decrease the activation of the RAGE pathway, potentially the lowering systemic inflammatory responses that are elevated in diabetic patients [18]. SI also contributes to enhanced glycemic control by reducing inflammation and the burden of AGEs, which can contribute to better glycemic control in diabetic patients, as chronic inflammation is known to exacerbate insulin resistance [17,18]. Moreover, AGEs are known to induce systemic inflammation through the activation of pro-inflammatory cytokines such as TNF-α and IL-6. Periodontal therapy, including SI, has been associated with a reduction in the systemic levels of these cytokines, thus reducing the overall inflammatory burden in diabetic patients [21,22]. Previous recent studies have shown that SI is effective in reducing the levels of HbA1c [35,36,37] in diabetic patients. Collectively, these studies demonstrated that SI in patients with periodontitis and DM helps reduce AGE accumulation, decreases the activation of inflammatory pathways, improves glycemic control, and lowers systemic inflammation, contributing to an overall improvement in periodontal and systemic health [38]. Conversely, SI is less successful in the CAL gain or inflammation reduction in DM patients with periodontitis when compared to systemic health patients with periodontitis. Therefore, this study aimed to evaluate the effect of NSPT on both the periodontal clinical parameters and glycemic metabolic control in patients with or without type 2 DM. The null hypothesis was that NSPT will not alter either the periodontal parameters or glycemic metabolic control.

## 2. Materials and Methods

### 2.1. Study Design

This non-randomized clinical trial was conducted at a single facility between September 2020 and January 2022. The study protocol was submitted to and approved by the Research Ethics Committee of the Faculty of Dentistry of Araçatuba (CAAE: 15049819.1.0000.5420). It was also registered with the Brazilian Registry of Clinical Trials (RBR-777nzpz) on the International Clinical Trials Registry Platform (U1111-1299-1687), following the guidelines of the CONSORT Statement for clinical trials.

### 2.2. Sample Calculation

Based on previous studies [39,40], the sample size was calculated to achieve 80% power (α = 5%; type B error = 20%) for detecting a significant difference of 1 mm in the PPD between the experimental groups, assuming a standard deviation of 0.90 mm. Consequently, a total of 15 patients were required. To account for a 20% attrition rate, 20 patients per group were included.

### 2.3. Sample Selection

A total of 40 individuals, aged 36 to 70 years, were included in this study. Twenty participants with poorly controlled type 2 DM (HbA1c ≥ 7.0%) and periodontitis were assigned to Group DM/P, while 20 participants with periodontitis but without DM (HbA1c ≤ 6.5%) were assigned to Group P.

Inclusion criteria were as follows: individuals of both sexes aged 30 to 70 years; diagnosis of stage II, III, or IV periodontitis [10]; no periodontal treatment within the last 3 months; a minimum of 15 teeth, excluding third molars; and, for Group DM/P, a diagnosis of type 2 DM (HbA1c ≥ 7.0%), while for Group P, no diagnosis of type 2 DM (HbA1c ≤ 6.5%).

The exclusion criteria were as follows: former smokers; individuals with anemia; those with active cancer or a history of chemotherapy; a history of antibiotic or anti-inflammatory therapy within the last 6 months; blood disorders; pregnancy; chronic kidney disease; those currently undergoing orthodontic treatment; individuals requiring prophylactic antibiotic therapy [41]; and osteoporosis under/without therapy with bisphosphonates.

The participants were recruited from the Periodontics Clinic of the School of Dentistry at Araçatuba—UNESP. Prior to enrollment, the patients were thoroughly informed about the etiology of periodontitis and given OHI tailored to their needs, including the use of toothbrushes, dental floss, and interproximal brushes. Those who expressed interest in participating signed an informed consent form.

### 2.4. Examiner Calibration

Before the experimental phase, an intra-examiner calibration was performed on 2 individuals, with 170 sites evaluated. Duplicate measurements of the PPD and the CAL were taken on two separate occasions, one week apart. The intra-examiner agreement for the PPD and the CAL was assessed using the Kappa test, yielding a value of 0.88, indicating substantial agreement.

### 2.5. Experimental Design and Treatment

The initial treatment for periodontitis consisted of NSPT combined with OHI. The participants underwent a 2 h session of SI using an ultrasonic device (Dabi Atlante, Ribeirão Preto, SP, Brazil) and manual Gracey and McCall curettes (Hu-Friedy, Chicago, IL, USA), in accordance with clinical practice guidelines [32]. All the SI procedures were performed by a single, experienced periodontist.

One week after SI, the participants were visually inspected for any adverse signs or symptoms. Follow-up visits were conducted at 90 and 180 days, during which the clinical examinations were repeated, and laboratory tests were conducted using the same parameters as at baseline. During these follow-up visits, supragingival plaque control and the OHI were reinforced according to each participant’s needs [14].

### 2.6. Primary and Secondary Clinical Outcomes

All the clinical periodontal parameters were measured using a millimeter periodontal probe (PCPUNC-15, Hu-Friedy Co., Chicago, IL, USA). A single, previously calibrated, blinded examiner performed the clinical examinations at baseline, 90, and 180 days post-treatment. The primary clinical outcome was a reduction in the PPD. Secondary outcomes included the number of teeth, the CAL gain, the BoP at all tooth sites (excluding third molars), the plaque index (PI) across four sites per tooth, and the HbA1c levels.

### 2.7. Clinical Analysis

The clinical data were tabulated, and the percentages of sites with the PPD and the CAL were categorized accordingly. The categorical data for the PI and the BoP were also converted into percentages, as previously described [41].

### 2.8. Glycemic Analysis

Glycemic control and the participants’ previous history of diabetes were assessed through a questionnaire (anamnesis) and the results of glycated hemoglobin tests, which were conducted at baseline and at 90 and 180 days after NSPT, were recorded.

### 2.9. Statistical Analysis

The demographic data, clinical periodontal parameters, and HbA1c results were organized using Microsoft Excel and subjected to descriptive and analytical statistical analyses with GraphPad Prism 6.0. A significance level of 5% was established. The null hypothesis was rejected if *p* ≤ 0.05. The data were tested for normality using the Shapiro–Wilk, D’Agostino & Pearson, and Kolmogorov–Smirnov tests. Since the data followed a normal distribution (*p* > 0.05), parametric tests were applied.

The clinical periodontal parameters and glycated hemoglobin levels were compared using a repeated measures ANOVA with Tukey’s post-hoc test across the baseline, 90 days, and 180 days. Comparisons between the different groups (Group DM/P vs. Group P) at each time point were evaluated using the *t*-test. 

## 3. Results

**Figure 1:**
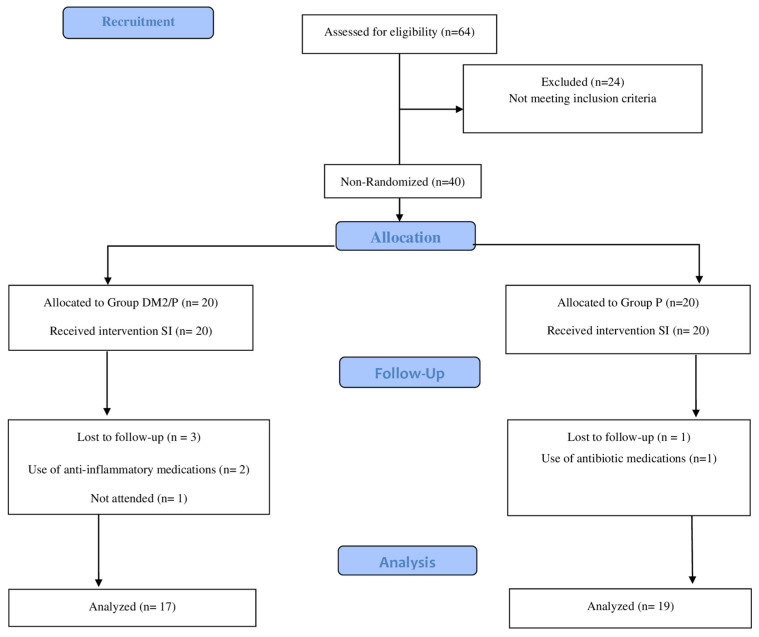
Flowchart of the study.

The results of this non-randomized clinical trial demonstrated that out of 64 participants considered for the study, 24 were excluded due to not meeting the eligibility criteria, leaving a total of 40 participants for the clinical study. In Group DM/P, three participants did not return for re-evaluation, resulting in a final sample size of 17. In Group P, one participant was excluded due to antibiotic use during the study, resulting in a final sample size of 19. Figure 1 illustrates the flowchart for the initial sample’s composition.

The additional comorbidities observed in Group DM/P included arterial hypertension, with around 65% of participants reporting the development of new conditions alongside their hyperglycemic status. The hypoglycemic drugs that were most used by the participants were Metformin (81.82%) and Xigduo XR (18.18%) in Group DM/P. The antihypertensive drugs most used by the participants were Losartan (63.3%) and Atenolol (15.40%). Regarding diuretics, 45.45% of the participants used Hydrochlorothiazide in Group P.

The sample composition analysis revealed demographic and social homogeneity across the groups, as detailed in Table 1. The participants’ ages ranged from 36 to 70 years. The mean age in Group DM/P was 50.94 ± 11.55 years, whereas Group P had a mean age of 59.32 ± 8.29 years (*p* = 0.01). Group P was older than Group DM/P, indicating a lower predisposition to diabetes and a longer duration of periodontal destruction in Group P compared to Group DM/P, which developed the disease earlier and more severely. The predominant gender in Group DM/P was male, while Group P was predominantly female. Ethnically, most of the participants identified as white, followed by mixed race and, to a lesser extent, black. Educational levels were similar across groups, with most of the participants having incomplete secondary education and a minority holding a university degree.

Table 2 compares the periodontal clinical variables and HbA1c values at baseline, 90 days, and 180 days. There was no significant difference in the number of teeth between the groups at baseline (*p* > 0.05). However, Group DM/P exhibited greater tooth loss at 180 days (*p* < 0.05). For the PI values, Group P showed a reduction at 90 and 180 days (*p* < 0.05), whereas Group DM/P had no significant changes in the PI over time.

Regarding the BoP, Group DM/P showed an increase at 180 days, though not statistically significant (*p* > 0.05). Group P demonstrated significant improvement in the BoP at both 90 and 180 days compared to baseline (*p* < 0.05; Table 2).

There were fewer sites with the PPD ≤ 4 mm across both the groups at baseline, with no significant difference. However, Group P experienced an increase in these sites at 90 and 180 days. Significant differences in the percentage of sites with the PPD ≥ 5 mm were observed between the groups at baseline, 90, and 180 days (*p* < 0.0001), with Group DM/P showing higher percentages. Additionally, Group DM/P demonstrated an increase in the PPD ≥ 5 mm at 180 days (Table 2).

For the CAL ≤ 3 mm, no improvement was observed in Group DM/P at any time point. In contrast, Group P showed significant gains in the CAL at 90 and 180 days compared to baseline. Both groups showed an increase in the CAL for sites with the CAL 4–5 mm at 180 days, with Group P showing greater gains compared to Group DM/P at 90 days (*p* = 0.001). The percentage of sites with the CAL ≥ 6 mm was reduced in Group P at 90 and 180 days compared to baseline. Group P had a higher percentage of CAL gain in sites with the CAL ≥ 6 mm compared to Group DM/P at baseline (*p* < 0.0001), 90 days (*p* < 0.01), and 180 days (*p* < 0.001). Group P also had a higher CAL gain for the CAL ≤ 3 mm compared to Group DM/P at 90 and 180 days (*p* < 0.0001; Table 2).

Among the diabetic patients, all of the participants had inadequate metabolic control, with HbA1c values greater than 7%. No significant differences were found within the groups over time, but significant differences were observed between the groups (*p* < 0.0001; Table 2).

**Figure 2:**
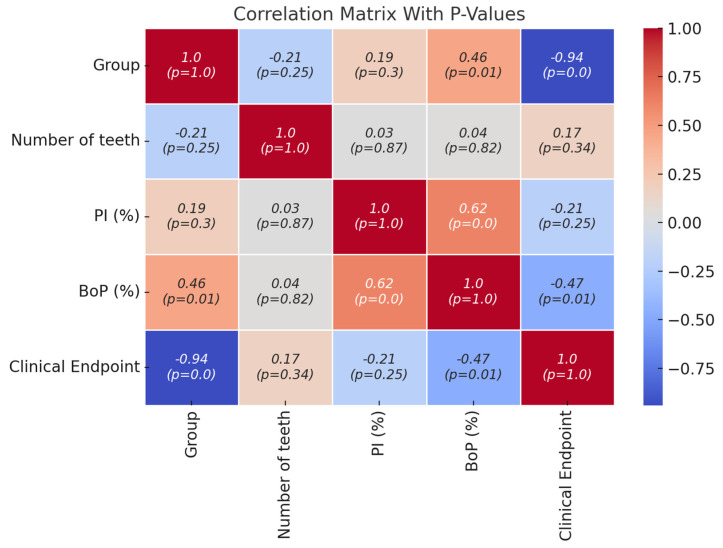
The Pearson correlation matrix between the predictor variables and the clinical endpoint. The Pearson correlation matrix between the variables, namely group, the number of teeth, the plaque index (PI), the bleeding on probing (BoP), and the clinical endpoint. The correlation values are accompanied by their respective *p*-values, indicated in parentheses. The intensity of the colors in the matrix reflects the strength and direction of the correlations, where colors closer to blue indicate negative correlations, and colors closer to red indicate positive correlations. Statistically significant correlations (*p* < 0.05) indicate important associations between the analyzed variables.

Figure 2 presents the Pearson correlation matrix for the analyzed variables, including the group, number of teeth, PI, BoP, and clinical outcome, i.e., the clinical endpoint [42]. The correlations, accompanied by p-values, indicate statistical significance. A strong negative correlation was observed between the group and the clinical endpoint (r = −0.94; *p* < 0.001), suggesting that group membership was a key determinant of clinical outcomes. A moderate negative correlation was found between the BoP and the clinical endpoint (r = −0.47; *p* = 0.01), indicating that higher BoP are associated with a lower likelihood of achieving the clinical endpoint. Conversely, the number of teeth (r = 0.17; *p* = 0.35) and the PI (r = −0.21; *p* = 0.25) showed weak, non-significant correlations with the clinical endpoint, suggesting a limited influence on the outcome. These results highlight that the group and the BoP were the main predictors of the clinical outcome, whereas the number of teeth and the PI had a minimal association.

Table 3 demonstrates the number of patients that reached the clinical endpoint, established with ≤4 sites with a PPD ≥ 5 mm after treatment. The results demonstrated that after 90 days 14 patients out of 19 achieved the endpoint. Similarly, after 180 days post-operation, 16 patients out of 19 reached the desired endpoint. Conversely, none of the participants in Group DM/P achieved the established endpoint of the study.

## 4. Discussion

Periodontitis and DM are chronic diseases with a high global prevalence [43]. DM is characterized by chronic hyperglycemia [44], while periodontitis is a multifactorial chronic inflammatory disease that is associated with a dysbiotic biofilm, leading to the progressive destruction of the supporting periodontal structures [10]. Both conditions can trigger inflammatory immune responses locally and systemically. DM is considered a risk factor for periodontitis, influencing its progression. Conversely, a hyperglycemic status (HbA1c ≥ 7) can accelerate periodontitis progression [45], making this association bidirectional. Therefore, the aim of this study was to investigate the clinical periodontal parameters in patients with DM following NSPT. Our findings indicate that DM impaired the clinical periodontal parameters, such as the BoP, the PI, and the PPD, and diminished the CAL gain. Our data suggest that DM effectively deteriorates periodontal tissue and aggravates the severity of the PPD and the CAL after 6 months post-operation.

The interventions for periodontitis typically include OHI, SI, and in some cases, systemic or local antibiotics [46]. Mechanical debridement (subgingival instrumentation) remains the gold standard for maintaining periodontal health. However, deep periodontal pockets, furcation lesions, and areas of limited assess may require adjunctive treatments to effectively manage the disease. Systemic antibiotic therapy, such as low-dose doxycycline combined with SI, has been shown to statistically reduce HbA1c levels in diabetic patients [47]. In this study, SI was performed without adjunctive therapies to strengthen the current evidence on the exclusive impact of DM on clinical periodontal parameters.

While some studies indicate that NSPT improves glycemic control in patients with type 2 DM and generalized chronic diseases [48,49,50], others studies report conflicting results, including an increase in HbA1c levels post-NSPT [51,52,53]. This study’s findings align with these discrepancies, as no significant improvement in HbA1c levels was observed in-Group DM/P at 90 or 180 days, though a modest average reduction of 0.23% was noted at 90 days, partially rejecting the null hypothesis. Meta-analyses on the effects of periodontal treatment on glycemic control in diabetic patients also show mixed results due to various metabolic changes [51,53]. The bidirectional relationship between DM2 and periodontitis suggests that type 2 DM increases the risk of periodontitis in poorly controlled patients and that periodontal inflammation can adversely affect glycemic control [54,55]. This study underscores the importance of this relationship. Potential explanations for the study’s findings include patient non-compliance with biofilm control, psychosocial factors exacerbated by the COVID-19 pandemic, and uncontrolled variables such as changes in hypoglycemic medication use, dietary habits, and overall health management. Despite stable HbA1c levels during follow-up, NSPT aided in stabilizing glycemia.

The periodontal parameters in Group DM/P were less favorable compared to Group P, which showed significant improvement after NSPT. Group P presented participants that were older compared to the DM/P individuals, who were associated with advanced gingival recession, which resulted in a higher percentage of sites with the CAL > 6 mm, indicative of advanced periodontitis. Group P also exhibited a greater reduction in deep pockets and a higher percentage of sites with the CAL ≤ 3 mm, indicating improved clinical attachment levels. Both groups experienced a loss of clinical attachment in sites with the CAL 4–5 mm by 180 days.

Patients with an uncontrolled hyperglycemic status experience systemic complications due to chronic hyperglycemia, which exacerbates the formation of AGEs and affects immune response and tissue integrity [56]. The bidirectional association between type 2 DM and periodontitis was evident, as DM exacerbates periodontitis and periodontal inflammation adversely affects glycemic control [23]. Some studies suggest that NSPT can improve oral health and have positive effects on metabolic control, including glycemic and lipid metabolism, and systemic inflammation in type 2 DM patients. However, other studies, like the work conducted by Baeza et al. [29], have found NSPT to have a minimal impact on metabolic control and systemic inflammation. Mohan et al. [57] reported that SI led to greater changes in C-reactive protein (CRP) levels in patients with type 2 DM, emphasizing the marked systemic changes in this group. The discrepancies observed between our results and those in the literature may be influenced by a combination of factors, including sample characteristics, glycemic control, assessment methods, and additional interventions. A careful analysis of these aspects is crucial to understanding the reasons behind these differences and to guiding future research and clinical practices. The integration of multidisciplinary approaches, along with the greater standardization of assessment methods, may be essential for aligning future outcomes with the existing evidence in the literature.

Chen et al. [58] observed a significant association between improved periodontal statuses and decreased CRP levels, suggesting that NSPT contributes to a better systemic inflammatory status and reduces the risk of microvascular complications in diabetics. The present study found significant reductions in the PI at 90 and 180 days in Group P. Conversely, Group DM/P showed higher PI values, reflecting greater difficulty in maintaining plaque control, which is consistent with findings by Khader et al. [59], Shanmukappa et al. [60], and Bissong et al. [61], who reported worse oral hygiene in diabetic patients compared to non-diabetic patients. However, Sandberg et al. [62] found no significant differences in daily tooth brushing practices between diabetic and non-diabetic patients, though non-diabetic patients had slightly better interproximal hygiene. Jiang et al. [63] reviewed the impact of health plans and services during the pandemic, highlighting increased patient costs and healthcare spending, which may further challenge the adherence to treatment.

It is important to acknowledge the limitations of our study. First, the sample size of 20 patients per group is relatively small, which limits the generalizability of the findings to a broader population. Consequently, further clinical studies with larger sample sizes or longitudinal studies are needed to draw more definitive conclusions regarding the effects of SI on HbA1c levels and the impact of type 2 diabetes on the outcomes of NSPT. Moreover, the follow-up period was somewhat short, and more studies with increased periods of follow-up are indicated to confirm the current findings.

## 5. Conclusions

This study concludes that patients with poorly controlled type 2 DM exhibit worse bacterial plaque control, a greater progression of periodontitis, and an increased severity of periodontitis compared to non-diabetic patients. Additionally, the response to initial NSPT was less favorable in diabetic patients, with no significant impact on glycemic control.

## Figures and Tables

**Table 1:** Demographic characteristics of the sample.

Demographic Characteristics	Group DM/P *n* = 17	Group P *n* = 19	*p*-Value
Age—Mean (±SD)	50.94 (±11.55)	59.32 (±8.29)	0.016
Gender—*n* (%)			
Male	9 (52.94%)	8 (47.06%)	NS
Female	8 (42.11%)	11 (57.89%)	

Abbreviations: SD, standard deviation; NS, not significant. Bold font, *p*-value < 0.05. A comparison between the numerical variable (age) was made using the t-test. To analyze the categorical variable (sex), the chi-square test was used.

**Table 2:** Comparison of periodontal clinical parameters [mean (± SD)] and HbA1c levels evaluated at follow-up periods.

Parameter	Baseline	90 Days	180 Days
Tooth number			
Group DM/P	22.81 (±5.09) a	22.19 (±5.17) a	21.63 (±4.98) b
Group P	23.41 (±4.11) a	23.35 (±4.04) a	23.29 (±4.01) a
PI (%)			
Group DM/P	41.84 (±30.15) a	27.76 (±28.04) a	27.16 (±21.08) a
Group P	38.65 (±27.46) a	15.06 (±6.77) b	20.71 (±15.31) b
BoP (%)			
Group DM/P	29.15 (±11.93) a	22.50 (±14.52) a	35.46 (±26.90) a
Group P	45.90 (±15.48) a	20.97 (±9.65) b	17.90 (±8.37) b
PPD ≤ 4 mm (%)			
Group DM/P	70.05 (±25.27) a	62.75 (±17.98) a	55,96 (±19.86) a
Group P	93.46 (±6.37) a	98.92 (±1.55) b	99.07 (±1.75) b
PPD ≥ 5 mm (%)			
Group DM/P	32.09 (±19.83) a	34.41 (±19.57) a	43.23 (±19.51) b
Group P	6.54 (±6.37) a****	1.08 (±1.55) b****	0.93 (±1.75) b****
CAL ≤ 3 mm (%)			
Group DM/P	31.34 (±18.73) a	33.54 (±29.31) a	23.07 (±19.38) a
Group P	6.64 (±5.92) a****	1.10 (±1.59) b****	1.02 (±1.89) b****
CAL 4–5 mm (%)			
Group DM/P	58.05 (±17.50) a	56.04 (±21.60) a,b	67.81 (±18.25) b
Group P	65.86 (±16.36) a	75.06 (±14.24) a**	76.12 (±15.25) b
CAL ≥ 6 mm (%)			
Group DM/P	10.59 (±5.04) a	11.58 (±12.40) a	9.11 (±7.54) a
Group P	27.50 (±12.58) ****	23.84 (±14.45) *	22.85 (±14.49) **
HbA1c			
Group DM/P	9.01 (±2.09) a	8.78 (±2.42) a	8.89 (±2.12) a
Group P	5.74 (±0.51) ****	5.86 (±0.45) ****	5.82 (±0.55) ****

Abbreviations: SD, standard deviation; PI, plaque index; BoP, bleeding on probing; PPD, probing pocket depth, CAL, clinical attachment level; HbA1c, glycosylated hemoglobin; ^a,b^ = different letter means a statistically significant difference between the evaluated periods of the same group (*p* < 0.05, repeated measures ANOVA test with Tukey’s post-hoc test). Comparisons between groups in the same period using the *t*-test; * *p*-value between 0.01 and 0.05; ** *p*-value between 0.001 and 0.01; and **** *p* < 0.0001.

**Table 3:** The analysis of the number of patients who reached the clinical endpoint of ≤ 4 sites with a PD ≥ 5 mm after treatment.

	Group DM/P	Group P	*p*-Valor
Variables	*n* = 17	*n* = 19	**
Clinical endpoint, N (%)			
Baseline	0 (0)	0 (100)	1.000
90 days	0 (0)	14 (73.7%)	≤0.0001
180 days	0 (0)	16 (84.2%)	≤0.0001

** *p*-value of the comparison between groups in each evaluation period (Fisher test).

## Data Availability

The data generated in this research project are available for access by contacting the last author of this paper via email. They are stored electronically, as Excel worksheets.
